# Complete Genome Sequence of the Type Strain *Corynebacterium epidermidicanis* DSM 45586, Isolated from the Skin of a Dog Suffering from Pruritus

**DOI:** 10.1128/genomeA.00959-15

**Published:** 2015-08-20

**Authors:** Christian Rückert, Janine Eimer, Anika Winkler, Andreas Tauch

**Affiliations:** a Department of Biology, Massachusetts Institute of Technology, Cambridge, Massachusetts, USA; b Institut für Genomforschung und Systembiologie, Centrum für Biotechnologie (CeBiTec), Universität Bielefeld, Bielefeld, Germany

## Abstract

The complete genome sequence of *Corynebacterium epidermidicanis* DSM 45586 comprises 2,692,072 bp with 58.06% G+C content. The annotation revealed 2,466 protein-coding regions, including genes for surface-anchored proteins with Cna B-type or bacterial Ig-like domains and for an adhesive SpaABC-type pilus with similarity to fimbrial subunits of *Corynebacterium resistens* DSM 45100.

## GENOME ANNOUNCEMENT

Currently, the genus *Corynebacterium* comprises 97 species that were isolated from diverse environments (1–7). Some *Corynebacterium* species are used in industrial applications and food production, whereas others are commensals or well-known pathogens of humans and animals, including mammals and birds. A few species originally recovered from animals have been implicated in the transmission of zoonotic infections to humans. These infections occurred in healthy individuals with close contact to wild or companion animals (e.g., by dog bites) (8). *Corynebacterium auriscanis* was isolated from a localized dog bite infection in a previously healthy female (9). Likewise, *Corynebacterium freiburgense* was cultured from a wound swab of a female who had been bitten by her dog in her forearm (10). *Corynebacterium canis* was also isolated from a patient’s wound caused by a dog bite (11). A *Corynebacterium* species isolated from the skin of a dog is *Corynebacterium epidermidicanis*, represented by the type strain DSM 45586 (410^T^) (12). Here, were present the complete genome sequence of *C. epidermidicanis* DSM 45586 to provide insights into the gene repertoire of this corynebacterium from a dog with pruritus.

Purified genomic DNA of *C. epidermidicanis* DSM 45586 was obtained from the Leibniz Institute DSMZ (Braunschweig, Germany) and used as input for the construction of two DNA sequencing libraries. A whole-genome shotgun library was generated with the TrueSeq DNA PCR-free library preparation kit (Illumina), and a 7-kb mate pair library was prepared with the Nextera mate pair sample preparation kit according to the gel-plus protocol (Illumina). The whole-genome shotgun library was sequenced in a paired-end run using the MiSeq reagent kit version 3 (600 cycles) and the MiSeq desktop sequencer (Illumina). The shotgun sequencing approach yielded 1,582,565 paired reads and 266,953,870 detected bases. An initial assembly of the paired reads was performed with the Roche GS *De novo* Assembler software (release 2.8) and resulted in 25 scaffolds, including 32 scaffolded contigs. The mate pair library was prepared for DNA sequencing with the MiSeq reagent kit version 3 (600 cycles), generating 692,492 mate pair reads that were added to the initial genome assembly to obtain a single scaffold. Gaps in the genome sequence were closed *in silico* with the Consed version 26 software package (13). Gene prediction was performed with the Prodigal software (14), and the functional annotation of the detected coding regions was carried out by the IMG/ER pipeline (15).

The chromosome of *C. epidermidicanis* DSM 45586 has a size of 2,692,072 bp with a mean G+C content of 58.06%. The fully automated annotation of the complete genome sequence revealed 12 rRNA genes, 52 tRNA genes, 12 other RNA genes, and 2,466 protein-coding regions, including 1,903 protein-coding genes with functional predictions. Cell-cell contact of *C. epidermidicanis* DSM 45586 with the animal host is probably mediated by surface-anchored proteins with Cna B-type or bacterial Ig-like domains (16, 17). A predicted pilus gene cluster consists of the *spaACB* genes with deduced amino acid sequence similarity to the subunits of the SpaABC pilus from *Corynebacterium resistens* DSM 45100 (18, 19).

### Nucleotide sequence accession number.

This genome project has been deposited in the GenBank database under the accession number CP011541.
